# Intravenous immunoglobulin bridging to rituximab in NMDAR encephalitis patients non-responders to first-line treatments

**DOI:** 10.1007/s10072-022-06313-3

**Published:** 2022-08-11

**Authors:** Federico Massa, Diego Franciotta, Stefano Grisanti, Luca Roccatagliata, Silvia Morbelli, Sabrina Beltramini, Antonio Uccelli, Angelo Schenone, Luana Benedetti

**Affiliations:** 1 Department of Neuroscience, Rehabilitation, Ophthalmology, Genetics, Maternal and Child Health (DINOGMI), University of Genoa, Largo Daneo 3, 16132 Genoa, Italy; 2 Autoimmunology Laboratory, IRCCS Ospedale Policlinico San Martino, Genoa, Italy; 3 IRCCS Ospedale Policlinico San Martino, Genoa, Italy; 4 Department of Health Sciences (DISSAL), University of Genoa, Genoa, Italy; 5 Pharmacy Complex Unit, IRCCS Ospedale Policlinico San Martino, Genoa, Italy

**Keywords:** Autoimmune diseases, Intravenous immunoglobulins, Modified-Rankin scale, NMDAR encephalitis, Rituximab, CASE score

## Abstract

**Background:**

The immunotherapy strategy for autoimmune encephalitis is based on several types and schedules of both first- and second-line drugs. Failing to respond to the latter prompts the use of non-conventional rescue therapies, with higher risks of severe adverse effects. We report on a protocol that entails the use of intravenous immunoglobulin cycles to bridge the 4-month period that the second-line drug rituximab needs to exert its full therapeutic effects.

**Methods:**

Three patients with NMDAR encephalitis who were non-responders to first-line treatments entered the study. The protocol consisted of six monthly cycles of intravenous immunoglobulins (IVIG, 0.4 mg/kg/die for 5 days), starting 1 month after the last rituximab infusion (1000 mg at days 0 and 15). Brain MRI and [^18^F]-FDG-PET were performed at onset and at six and 18 months after onset.

**Results:**

In the three patients, substantial improvements of disability or complete recovery were achieved, without modifications over the 30-to-50-month follow-up. No adverse events nor laboratory test abnormalities were recorded. Imaging findings paralleled the favorable disease courses. Brain [^18^F]-FDG-PET was more sensitive than MRI in detecting abnormalities.

**Discussion:**

Our observations suggest that the herein-described protocol might be used in patients with NMDAR encephalitis at risk for poor prognosis in the mid-term when they need to shift to rituximab. [18F]-FDG-PET confirmed to be a sensitive tool to detect the minimal brain lesions that can underlie isolated cognitive and psychiatric symptoms.

## Introduction

NMDA receptor (NMDAR) encephalitis is a rare autoimmune disease whose prognosis is highly influenced by timely recognition and treatment [1]. Immunotherapeutic approaches are heterogeneous regarding (a) type and schedule of both first- and second-line drugs and (b) criteria for evaluating their efficacy and deciding when and how to escalate [2]. There is enough evidence supporting the value of the B-cell-depleting rituximab in NMDAR encephalitis in terms of good functional outcome and balanced safety profile [3]. Switching to this drug could be considered when one or more first-line treatments are poorly effective, or to reduce the risk of relapses [2]. When even the response to rituximab is unsatisfying, some non-conventional rescue therapies, such as protease-inhibitor bortezomib and anti-IL6 receptor tocilizumab, have been seldom reported beneficial [4–7]. Given the caveats for risk–benefit profiles of these drugs, substantial uncertainty exists on how to identify rituximab-resistant patients and when to shift. For example, “no clinical improvement 1 month after the last administration” might seem a pragmatic approach to promptly controlling the disease, but this strategy hampers the assessment of the delayed effects of rituximab [5, 6]. Indeed, it is well-established that rituximab depletes B cells from the circulation 1 month after each cycle [8], and substantial clinical responses occur over 4 months after the first infusion in autoimmune encephalitis [9]. Misinterpreting rituximab inefficacy implies risks of overtreatments and safety.

## Patients and methods

To cover the period that rituximab needs to take effect and limit the switch to possibly unnecessary rescue treatments, our protocol entailed six monthly cycles of intravenous immunoglobulins (IVIG, 0.4 mg/kg/die for 5 consecutive days), starting from the month after the last rituximab infusion (1000 mg at days 0 and 15). The therapeutic protocol was used in three patients with definite NMDAR encephalitis [10] non-responders to first-line treatments, thus at risk of long-term poor functional outcomes. In one case (Pt #3), an ovarian teratoma was associated with NMDAR encephalitis. We conducted quarterly clinical follow-up with assessment of severity (Clinical Assessment Scale in autoimmune Encephalitis, CASE) [11] and disability (modified-Rankin scale, mRS) for 30-to-50 months, and extensive hematology and clinical chemistry evaluations (including CD19 + , CD20 + , and CD27 + B-cell counts) every 6 months. Brain MRI and [^18^F]-FDG-PET were performed at onset and after six and 18 months from onset. An expert radiologist (L.R) and a nuclear medicine physician (S.M.) visually assessed the brain MRI and [^18^F]-FDG-PET scans, respectively.

All subjects gave their consent to use anonymized data for this study, approved by the local Ethics Committee, and conforming with the Declaration of Helsinki.

## Results

**Fig. 1:**
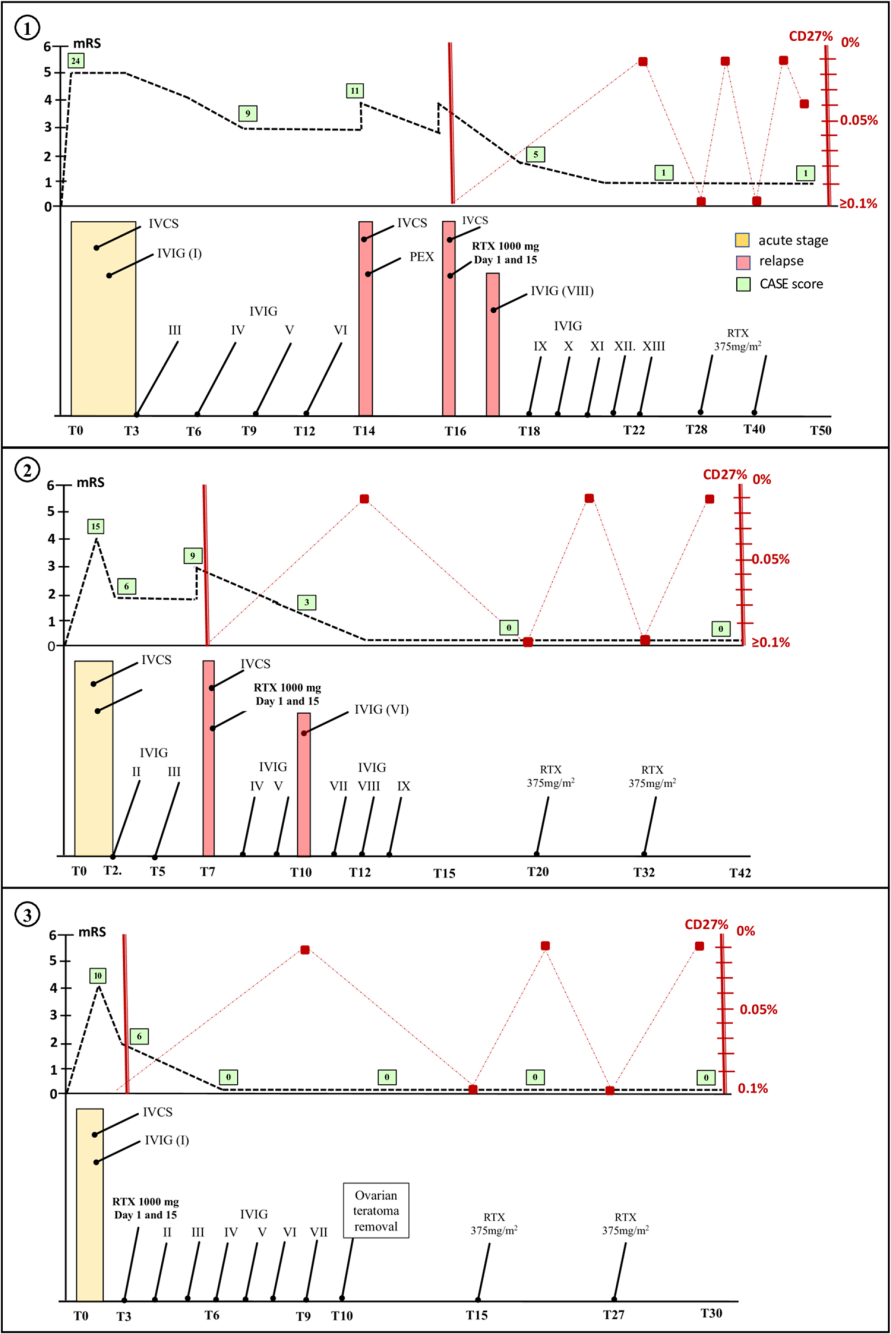
Immunotherapies administered and longitudinal clinical and CD27 + B-cell assessments in the three patients with NMDAR encephalitis. Acute stage (yellow boxes) and clinical relapses (light red boxes); disability was measured with modified-Rankin scale (mRS) scores (left *Y*-axis) and CASE score (light green numbered boxes) at the various time points (*T*) (numbers, months from onset; patient’s number in the circle). Percentages of blood CD27 + B cells after the first rituximab infusion and during follow-up (right *Y*-axis). CASE, Clinical Assessment Scale in autoimmune Encephalitis; IVCS, intravenous corticosteroids; IVIG, IV immunoglobulins; PEX, plasma exchange; RTX, rituximab

**Fig. 2:**
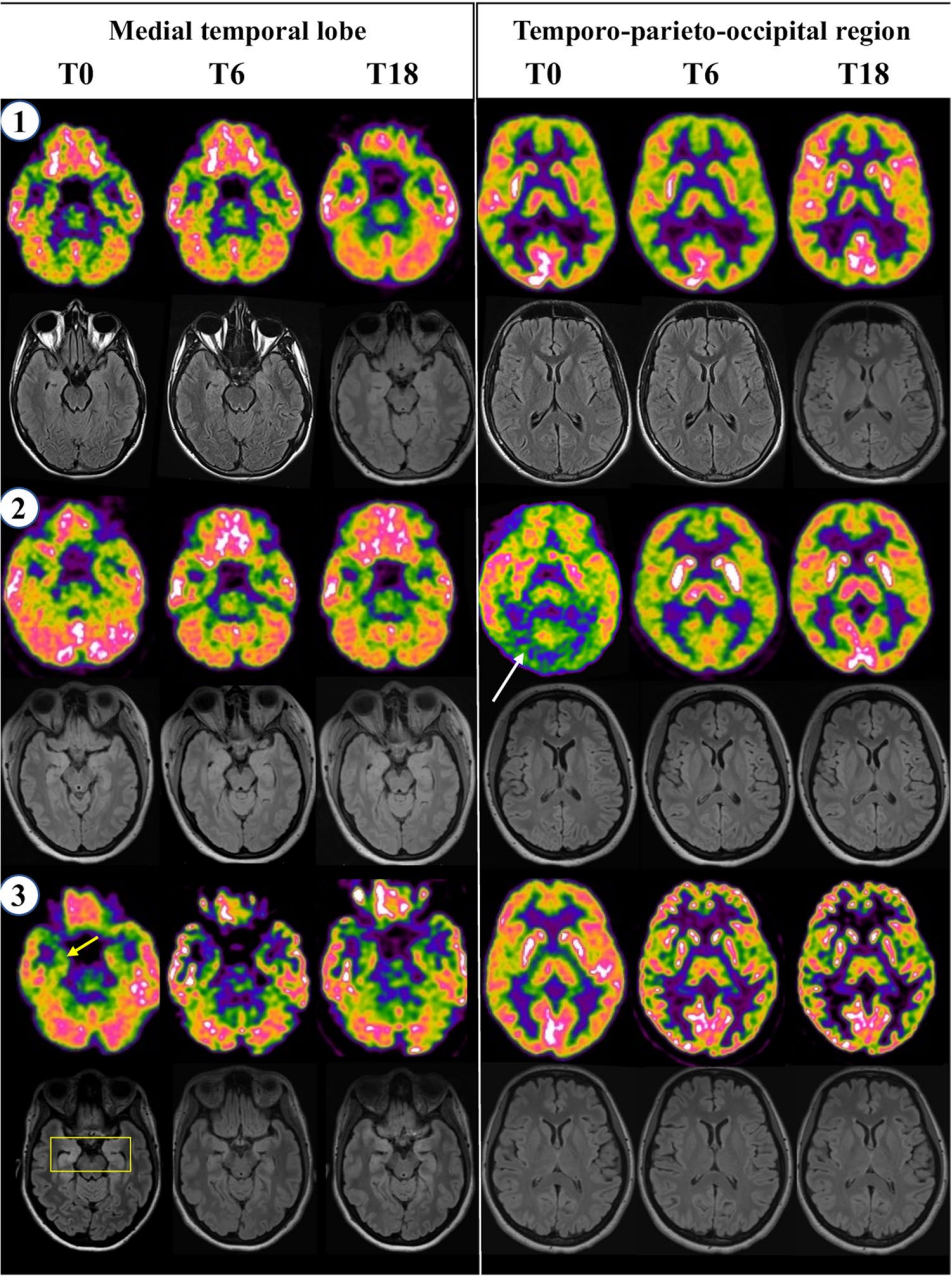
Longitudinal imaging findings in the three patients with NMDAR encephalitis. [^18^F]-FDG-PET (upper parts of the panels) and matching MRI-T2-Flair sequences (lower parts) (patient’s number in the circle). Axial scans display the medial temporal lobe (on the L) and temporo-parieto-occipital region (on the R). MRI showed slight hyperintensity in medial temporal lobes in patient #3 only at T0 (R > L, yellow rectangle), whereas [^18^F]-FDG-PET showed (a) hypometabolism in lateral temporal and posterior parietal lobes bilaterally, and asymmetry (L < R) in frontal-insular and thalamic regions; the abnormalities were more evident at T6, almost entirely solved at T18, with only a slight asymmetry in the frontal-insular cortex and thalamus (patient #1); (b) bilateral occipital hypometabolism at T0 (white arrow), and relative slight hypermetabolism in basal ganglia compared to widespread cortical hypometabolism at T6; only a slight asymmetry (L < R) remained in temporo-polar regions at T18 (patient #2). Metabolism in the medial temporal lobe was unremarkable in both patients #1 and #2 and reduced (R < L) in patient #3 (yellow arrow), matching the MRI-detected hyperintensity; this abnormality was utterly solved at T6-18. L, left; R, right; T, timepoint; numbers, months from onset

Table 1 summarizes the clinical and demographic features of the patients. Cerebrospinal (CSF) analysis was remarkable for pleocytosis and positivity of NMDAR antibodies (cell-based assay, Euroimmun, Germany). Figure 1 shows the longitudinal clinical assessment and immunotherapies at various time points. Briefly, the patients received IV corticosteroids (IVCS; methylprednisolone 1000 mg/day for 5-to-10 days) closely followed by IVIG infusions (0.4 mg/kg/die for 5 days). Treatment response was null or incomplete (see mRS scores at T3, Fig. 1). Poor clinical improvement in patients #1 and #2 led to repeat IVIG cycles. They relapsed after 14 and 7 months, respectively. Incomplete recovery (mRS score ≥ 2) prompted escalation to our treatment protocol. In patient #1, escalation was delayed because of septic arthritis that arose during intensive care unit (ICU) admission. Minor psychotic relapses occurred in patient #1 4 weeks after rituximab administration and in patient #2 after 10 weeks, without changes in mRS scores. Hence, the patients received the monthly IVIG as scheduled (Fig. 1). Maintenance rituximab infusions (375 mg/m^2^) were administered when CD27 + B-cell percentages exceeded 0.05% of peripheral blood mononuclear cells (approximately yearly). Substantial improvements of disability or complete recovery were achieved after 6 months, lasting unchanged over the entire follow-up. No adverse events nor laboratory test abnormalities, which were checked every 6 months during the entire follow-up, were recorded. Imaging findings paralleled the favorable disease courses (details in Fig. 2).

**Table 1:** Demographic and clinical findings

	Related tumor	Symptoms at onset	Symptoms of full-blown encephalitis	ICU	CSF WBC(OCB Y/N)	1^st^ line treatment	Onset to 1^st^ line (days)	Symptoms of first acute relapse	Onset to RTX (months)	Follow-up (months)	Short-term outcome (2–6 months)	Long-term outcome (> 24 months)
Pt #1 M, 17y	No	Perceptive hallucinations, psychomotor agitation	Memory and speech disorder; ULs and orofacial dyskinesias; dysautonomia; NORSE; coma	Y*	165/mm^3^ (Y)	IVCS, IVIG (× 2)	45	Secondary generalized seizures, psychomotor agitation	16	54	Deficit in working memory, speed processing, complex reasoning; anxiety and irritability; sociality issues	Deficit in complex reasoning, mild anxiety
Pt #2 F, 16y	No	Apathy, behavioral disorder	Cognitive disorder; speech impairment; auditory hallucinations; psychomotor agitation, motor stereotypies	N	80/mm^3^ (Y)	IVCS, IVIG (× 2)	32	Auditory hallucinations, psychomotor agitation, self-harm behavior	7	42	Deficit in attention and speed processing, anxiety, auditory hallucinations	Complete recovery
Pt #3 F, 38y	Ovarian teratoma	Short-term memory impairment, apathy	Behavior disorder; speech impairment psychomotor agitation; catatonia	N	25/mm^3^ (Y)	IVCS, IVIG	35	-	2	30	Deficit in short-term memory, anxiety	Complete recovery

*Pt*, patient; *M*, male; *F*, female; *y*, years; *ULs*, upper limbs; *NORSE*, new-onset refractory status epilepticus; *ICU*, intensive care unit admission; *Y*, yes; *N*, no; *CSF*, cerebrospinal fluid; *WBC*, white blood cell count; *OCB*, CSF-restricted oligoclonal bands; *IVCS*, intravenous corticosteroids (1000 mg for 5–10 days); *IVIG*, intravenous immunoglobulins (0.4 mg/kg/die for 5 days); *RTX*, rituximab (1000 mg at days 0 and 15); *50-day stay

## Discussion

This is the first description of a combined IVIG-plus-rituximab therapeutic scheme that allowed to reach long-term remission of NMDAR encephalitis in three patients non-responders to first-line treatments. The underlying rationale was to prevent disease relapses over the 6 months during which rituximab can be incompletely effective [9]. Two patients suffered only from non-disabling psychiatric symptoms in this period, as it often occurs in NMDAR encephalitis [1].

Delay in diagnosis, relapses, residual disability, and young age were the main elements inducing the escalation to monthly IVIG cycles bridging to rituximab after first-line treatments in our case series. Other adverse prognostic factors included brain MRI hyperintensity, CSF pleocytosis, and ICU admission.

Protocols combining IVIG and rituximab to treat autoimmune diseases are not new, especially in rheumatology, even with the idea that the combination might reverse autoimmunity. Indeed, the so-called “Ahmed Protocol” entails the use of IVIG cycles until rituximab-induced B-cell repopulation occurs, with additional cycles to end the treatment [12]. Reduction or disappearance of pathogenic autoantibodies matched clinical recovery, which induced the authors to claim that their protocol was able to suppress the inflammatory process in the microenvironment and then to restore immune tolerance in the long term thanks to IVIG cycles [12]. Their protocol substantially differs from our therapeutic scheme, which aims at harnessing the IVIG potential of passively clearing pathogenic autoantibodies and competing with them at antigenic target level, restoring tolerance by mimicking checkpoints, and decreasing the risk of infection [13, 14]. In addition, differently from rheumatoid arthritis, the pathogenesis NMDAR encephalitis is preeminently driven by the humoral immune response [15]. Therefore, IVIG cycles could maintain as lower as possible the circulating levels of NMDAR antibodies, whose pathogenic potential is directly associated with their ability to bind and functionally interfere with their antigenic targets [15]. Exerting this potential allows waiting confidently for the complete therapeutic expression of rituximab, which needs at least a 4-month period, and avoiding an early switch to other rescue treatments. On the contrary, the number of IVIG cycles of the “Ahmed Protocol” covers the entire timeframe occurring between the first rituximab infusion and repopulation of memory B cells [12].

Our therapeutic scheme led to excellent outcomes in the long term, regardless of pre-rituximab status or therapy escalation timing (2, 7, and 16 months after NMDAR encephalitis onset in the three patients). Notably, escalating to second-line treatment was later in our cases than in those described by other authors in anti-NMDAR encephalitis (median 54 days), but without substantial differences if compared to the timing reported in anti-LGI1, CASPR2, or GAD65 encephalitis (median 155, 632, and 1209 days) [9]. In Pt #1, the delay was due to post-ICU infectious complications, whereas in the others we decided for a wait-and-see strategy and to escalate to second-line treatment in case of incomplete recovery, either with (Pt #2) or without a clinical relapse (Pt #3). Notwithstanding that escalation was delayed, with the risk of reducing rituximab chances of efficacy, our treatment scheme resulted successful in the long-term remissions as full recovery in two patients and a substantial improvement of disability in the other were obtained. Moreover, and to further support our therapeutic scheme’s consistency, patient #3 showed full recovery even before the removal of her ovarian teratoma.

[^18^F]-FDG-PET was more sensitive than MRI in matching clinical courses, even at the level of cognitive and psychiatric symptoms, thus confirming to yield information usable as consistent as biomarkers of disease severity, recovery after treatment, and functional outcome [16, 17].

Although limited to a small case series and without a control group, but within a context of rare forms of autoimmune encephalitis possibly associated with bad prognosis and a non-negligible relapse rate [1], our study suggests that IVIG bridging to rituximab is safe and long-term effective in NMDAR encephalitis patients non-responders to first-line therapies. Because NMDAR encephalitis has a long-term recovery period, the possibility that long-term disease remission could rely on rituximab alone cannot be completely ruled out. On the other hand, bridging IVIG enabled prompt disease control with very mild symptoms in patients who had previously experienced disabling relapses. In the absence of data from randomized controlled trials, our therapeutic scheme represents a possible successful approach to NMDAR encephalitis forms likely characterized by poor prognosis, thus preventing possible premature escalation to more aggressive and less safe rescue treatments. Further studies, with a prospective design and including a larger sample size, are needed to establish the most effective treatment strategy in those forms of anti-NMDAR AE at risk for poor prognosis in the mid-long term.
